# Assessment of total hepatitis C virus (HCV) core protein in HCV-related mixed cryoglobulinemia

**DOI:** 10.1186/ar4513

**Published:** 2014-03-18

**Authors:** Sabino Russi, Domenico Sansonno, Maria Addolorata Mariggiò, Angela Vinella, Fabio Pavone, Gianfranco Lauletta, Silvia Sansonno, Franco Dammacco

**Affiliations:** 1 Liver Unit, Division of Internal Medicine and Clinical Oncology, University of Bari “Aldo Moro”, Bari, Italy; 2 Laboratory of General Pathology and Experimental Oncology, University of Bari “Aldo Moro”, Bari, Italy; 3 Department of Biomedical Sciences and Human Oncology, University of Bari “Aldo Moro”, Bari, Italy; 4 C.U.R.E. Centre for Liver Diseases Research and Treatment, Institute of Internal Medicine, Department of Medical and Surgical Sciences, University of Foggia, Foggia, Italy

## Abstract

**Introduction:**

In hepatitis C virus (HCV)-related mixed cryoglobulinemia (MCG), the nonenveloped HCV core protein (HCV-Cp) is a constituent of the characteristic cold-precipitating immune complexes (ICs). A possible correlation between HCV-Cp, virologic, laboratory, and clinical parameters in both untreated MCG patients and those undergoing specific treatment was explored.

**Methods:**

HCV-Cp was quantified by a fully automated immune assay. Correlations between HCV-Cp and HCV RNA, cryocrit, and virus genotype (gt) were investigated in 102 chronically HCV-infected MCG patients.

**Results:**

HCV-Cp concentrations strongly correlated with HCV RNA levels in baseline samples. An average ratio of 1,425 IU and 12,850 IU HCV RNA per picogram HCV-Cp was estimated in HCV gt-1 and gt-2 patients, respectively. This equation allowed us to estimate that, on average, HCV-Cp was associated with the viral genome in only 3.4% of the former and in 35% of the latter group of patients. The direct relation between HCV-Cp and the cryocrit level suggests that the protein directly influences the amount of cryoprecipitate. Although the therapy with rituximab (RTX) as a single agent resulted in the enhancement of HCV-Cp levels, in patients treated with RTX in combination with a specific antiviral therapy (pegylated interferon-α plus ribavirin), the prompt and effective clearance of HCV-Cp was documented.

**Conclusions:**

Our data provide evidence that HCV-Cp has a direct effect on the cold-precipitation process in a virus genotype-dependence in HCV-related MCG patients.

## Introduction

Chronic HCV infection is often associated with a large spectrum of B-cell abnormalities [1,2], the most prominent of which is the persistent production of circulating immune complexes (ICs) [3-5]. In almost one third of HCV-infected individuals, these ICs have cryoprecipitating properties [6]. Within the clinical spectrum of chronic HCV infection, mixed cryoglobulinemia (MCG), characterized by overt systemic vasculitis, nephropathy, and neuropathy, is a striking feature [7]. However, with or without MCG, HCV infection is associated with an increased risk of B-cell non-Hodgkin lymphoma (B-NHL) [8,9].

Restriction of both humoral and cellular immune responses, as defined by enrichment of B- and T-cell clonal expansions, has been detected in patients with MCG [10-12]. Dominant cell clones contribute to the formation of intraportal follicle-like structures in chronically HCV-infected livers [13]. Sequence analysis of the heavy-chain complementarity-determining region-3 (CDR-3), whether from circulating B-cell expanded clones or isolated from liver follicle-like structures, demonstrated numerous variations in this immunoglobulin gene segment, supporting the notion that these cells are the result of an antigen-driven response [14,15]. In addition, in a previous study, we determined that the restriction of B-cell V gene use has a direct clinical impact, in that it is associated with high serum levels of rheumatoid factor (RF) and extrahepatic disorders [13,16]. The mechanism(s) of B-cell stimulation, however, is not known, nor has it been established whether it is directly related to MCG. However, the answers to these questions are of interest, given that only a subset of individuals with chronic HCV infection develops MCG and/or NHL. Furthermore, in MCG, memory, but not naïve B cells are activated, and MCG-specific activation markers have been described [17].

The cold-dependent insolubility of ICs in HCV-related MCG is thought to result from a host reaction involving primarily IgM molecules with RF activity and capable of activating the complement cascade through the binding of the globular domain of the C1q receptor (gC1q-R) [18,19]. In certain viral infections, including HIV and EBV, the responding naïve and memory B cells produce polyreactive antibodies that exhibit bivalent heteroligation between a high-affinity combining site and a second low-affinity molecular site located elsewhere on the pathogen [20]. In MCG, conversely, HCV/IgG ICs are thought to engage with an RF-like B-cell receptor to stimulate B-cell expansion, which becomes autoreactive through antigen-dependent somatic hypermutations [21]. This pathogenetic model, however, has yet to be confirmed because the antigen(s) potentially involved in B-cell activation have not been identified.

The immunochemical structure of cold-precipitating ICs is still poorly known, and the role of HCV in cryoprecipitation has been shown only indirectly. We have also not determined whether and to what extent HCV envelope glycoproteins or defective interfering viral particles participate in cryoprecipitation. Nonetheless, in a previous study, we showed that HCV nucleocapsid (HCV-Cp), devoid of envelope proteins, is a constitutive component of cryoglobulins [22] and is potentially able to cause cryoglobulin-mediated tissue injury *in vivo,* via gC1q-R [23]. In this model, C1q acts as a bridging molecule between circulating ICs containing HCV-Cp and the vascular endothelium [18].

Tests for the quantitative determination of HCV-Cp have been introduced to supplement PCR-based analysis of HCV RNA [24,25], to monitor antiviral therapy [26,27], and to diagnose HCV infection in immunocompromised patients and in those undergoing hemodialysis [28]. Recently, a fully automated, highly sensitive chemiluminescent immunoassay was developed to overcome the shortcomings of conventional core assays [24,29]. In this study, we made use of this advanced method to examine the possible correlation between HCV-Cp, HCV RNA, and viral genotypes (gts) in relation to cryoglobulin production. The effect of B-cell depletion and specific antiviral therapy on the levels of circulating and cold-precipitated HCV-Cp was also explored.

## Methods

Included in the study were 102 consecutive, unselected patients with HCV-related MCG diagnosed between 2002 and 2012 at the Liver Unit, Department of Internal Medicine and Clinical Oncology, University of Bari Medical School. All patients had serum cryoglobulins associated with clinical signs consisting of purpuric vasculitis, arthralgias, and, inconsistently, nephropathy and/or neuropathy. The inclusion criteria were (a) positivity for anti-HCV antibodies and a positive PCR-based assay to detect HCV RNA in serum; (b) detection of serum cryoglobulins; (c) liver biopsy performed before enrollment; (d) negativity for HBsAg and HIV antibodies; and (e) no previous antiviral and/or immunosuppressive treatments. Baseline evaluation included demographic data, medical history, physical examination, evaluation of comorbidities, and use of medications. The study was carried out according to the principles of the Declaration of Helsinki and Good Clinical Practice and was approved by the Ethical Review Board of the University of Bari. Informed consent for the collection and storage of their serum and peripheral blood mononuclear cells (PBMCs) was obtained from all subjects.

### Laboratory parameters

Serum HCV antibodies were detected by second-/third-generation enzyme-linked immunosorbent assays (Abbott Lab, Chicago, IL, USA). Serum HCV RNA was determined by RT-PCR (Roche Diagnostics System, Branchburg, NJ, USA) and quantified with the Versant HCV RNA quantitative 3.0 assay (Siemens Healthcare, Erlangen, Germany). HCV genotyping was performed with INNO-LiPA (Innogenetics NV, Ghent, Belgium). Liver biopsy was carried out in all patients, and the histology specimens were scored for grade and stage according to the METAVIR score [30]. Neurologic assessment included strength evaluation, electromyography, motor and sensory nerve-conduction velocity, and short-latency somatosensory evoked potentials. Cryoglobulins were isolated as specified elsewhere [7]. Cryoprecipitates, diluted in 0.5 *M* NaCl, were fractionated with high-resolution gel electrophoresis, and monoclonal bands were identified by immunofixation. Cryoglobulins were classified according to Brouet *et al.*[31] as either type II, usually containing polyclonal IgG and a monoclonal IgM component, and type III, containing polyclonal IgG and IgM.

### HCV core protein determination

Cryopreserved serum samples, purified cryoglobulins, and supernatants were tested for HCV-Cp by using Architect HCV Ag (Abbott Diagnostics, Wiesbaden, Germany) and retested for quantitative HCV RNA with the Cobas Taqman assay (Roche Diagnostics GmbH, Mannheim, Germany). The Architect HCV Ag assay is a fully automated, quantitative, chemiluminescent microparticle immunoassay for the detection of nonenveloped HCV-Cp in HCV-infected sera [24]. One of the main feature of this assay is the automated on-board pretreatment step to lyse viral particles and to extract HCV-Cp by using solutions containing urea, hydrochloric acid, and detergents. In addition, the lysis step enables us to dissociate HCV-Cp from circulating ICs and, possibly, to detect noninfectious-containing HCV-Cp particles released from cells undergoing a necrotic process [26]. Total circulating nonenveloped HCV-Cp, regardless of its biologic and molecular status, is captured on the surface of paramagnetic microparticles coated with three anti-core monoclonal antibodies (moAbs) (that is, C11-3, C11-7, and AOT-3) recognizing different epitopes of core protein and revealed with other two acridium-labeled anti-core moAbs (that is, C11-9 and C11-14). The concentration of HCV-Cp in the specimen is determined by using a standard curve generated by running six calibrators in duplicate. The cut-off value of 0.06 pg recombinant C11 antigen (residues 1 to 160 of HCV gt-2a)/ml (3.0 f*M*) was set by the manufacturer.

The kinetics of serum HCV-Cp was explored in HCV-positive MCG patients under different therapeutic schedules. In schedule A, 16 patients unresponsive to a combination of pegylated interferon-α (pIFN-α) plus ribavirin (RBV) were given four intravenous infusions of rituximab (RTX; Hoffmann-La Roche Ltd., Basel, Switzerland), an anti-CD20^+^ chimeric monoclonal antibody, at a dose of 375 mg/m^2^ once a week over a period of 1 month. These patients were described in a previous study [32].

IN schedule B, eight patients received RTX as in schedule A, followed by pIFN-α once a week and RBV 800 to 1,200 mg/day according to body weight and viral gt.

The eight patients treated according to schedule C also received RTX, pIFN-α, and RBV, as in schedule B, albeit simultaneously.

### Statistical analysis

Differences between data were expressed as median and range. Spearman rank correlation coefficient (*r*) test was used to evaluate the relations between variables. Changes between measurements were assessed with nonparametric tests including Friedman test and KS test. The Mann–Whitney *U* test was used to compare HCV-gt-related variables. For all statistical analyses, IBM SPSS statistics version 19.0 was used. All reported *P* values were two-sided; *P* < 0.05 was considered significant.

## Results

All samples were analyzed for HCV-Cp twice with different lots of reagents. Analytic evaluation of 102 chronically HCV-infected patients with MCG was carried out by using five replicates of six cryoprecipitates with a low cryocrit (2.1% ± 0.6%): three samples from gt-1 patients, two from gt-2 patients, and one from a patient with gt-3. An additional six cryoprecipitates with a high cryocrit (11% ± 3.6%) were studied in parallel: three samples from gt-1 patients, two from gt-2 patients, and one from a patient with gt-3. The coefficient of variation (CV) was 3.16% for the low-cryocrit group and 3.76% for the high-cryocrit group.

**Figure 1:**
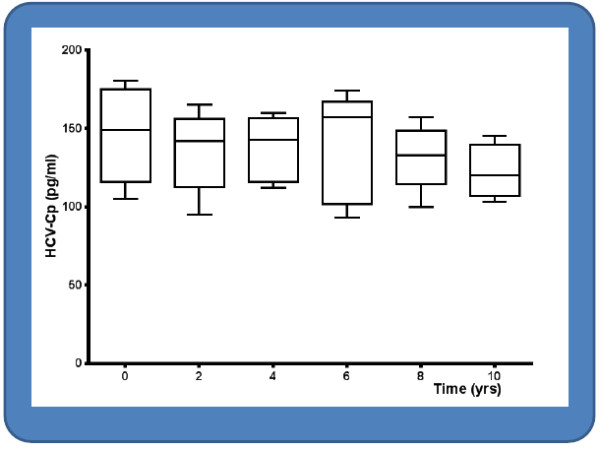
Time-related effect on the detection of nonenveloped HCV-Cp in the cryoprecipitate samples over a period of 10 years of storage.

Reproducibility was defined by a control study performed to verify the possible core protein shedding in the cryoprecipitates during a long storage period. Five patients who refused specific antiviral therapy and followed up for more than 10 years with low doses of steroids were evaluated. As reported in Figure 1, stable results were obtained over the considered long period. Furthermore, six specimens consisting of total serum and cold-precipitated samples of three HCV-positive MCG patients obtained in 2002 and three sera and cryoglobulins from three patients obtained in 2012 were assayed in replicates of two at two separate times per day by using two lots of reagents. Results showed a percentage CV of 6.5 and 4.6, respectively.

Specificity was tested by using the serum samples, cryoprecipitates, and supernatants of 19 HCV-negative MCG patients with the following clinical diagnoses: “essential” MCG, not associated with any apparent disease (five women and one man, median age of 59 years, ranging from 49 to 76, five with type II and one with type III MCG); connective tissue disorders [systemic lupus erythematosus (*n* = 3), dermatomyositis (*n* = 1), scleroderma (*n* = 1); four women and one man; median age of 60.5 years, ranging from 38 to 75 years, one with type II and four with type III MCG]; B-NHL (one woman and two men; median age of 52 years, ranging from 45 to 66 years, all with type II MCG); and chronic lymphocytic leukemia (two women and three men; median age, 58 years ranging from 51 to 78 years, all with type II MCG). Serum, cryoprecipitate, and supernatant samples obtained from each of these HCV-negative patients were consistently negative for HCV-Cp. The linearity of the assay was verified in five serially diluted cryoprecipitates with HCV-Cp levels >100 pg/ml. Linearity was maintained throughout the entire dynamic range; the r coefficient ranged between 0.92 and 0.97.

The epidemiologic, virologic, laboratory, and clinical characteristics are described in Table 1. The qualitative results, determined by HCV-Cp and HCV RNA assays, showed an agreement of 100%. All samples positive for HCV-Cp were also positive for HCV RNA. HCV-Cp was detected in all unfractionated serum specimens, with a median levels of 18.3 pg/ml, ranging from 1.3 to 175.6 pg/ml; in all cryoprecipitates with a median of 18.5 pg/ml ranging from 0.1 to 105 pg/ml; and in all supernatants with a median of 3.1 pg/ml, ranging from 0.2 to 91.3 pg/ml.

**Table 1:** Virologic, epidemiologic, histologic, laboratory, and clinical characteristics of 102 patients with HCV-related cryoglobulinemic vasculitis

**Virology**	
**Serum HCV RNA,***n* (%)	**102** (100)
**• Titer,** log IU/ml, median (range)	**5.64** (4.0-7.0)
**Serum HCV core protein,***n* (%)	**102** (100)
**• Titer,** pg/ml, median (range)	**18.3** (1.3-175.6)
**Cryoprecipitate core protein, n** (%)	**102** (100)
**• Titer,** pg/ml, median (range)	**18.5** (0.1-105)
**Supernatant core protein,***n* (%)	**102** (100)
**• Titer ,** pg/ml, median (range)	**3.1** (0.2-91.3)
**HCV Genotype,***n* (%)	
**• gt-1**	**51** (50)
**• gt-2**	**49** (48.1)
**• gt-3**	**2** (1.9)
**Anti-HCV antibodies,***n* (%)	**102** (100)
**HBsAg,***n* (%)	**0**
**Anti-HIV antibodies,***n* (%)	**0**
**Epidemiology**	
**Sex: M/F** (ratio)	**22/80** (0.27)
**Age,** years, median (range)	**68** (44–80)
**Presumed duration of cryoglobulinemic vasculitis,** years, median (range)	**7.5** (1–26)
**Blood/blood product transfusion,***n* (%)	**24** (23.5)
**Liver histology**	
**Chronic active hepatitis,** n (%)	**92** (90.2)
**Inflammation index,** median (range)	**3.1** (2.8-4.4)
**Fibrosis index,** median (range)	**2.2** (1.6-3.8)
**Cirrhosis,***n* (%)	**10** (9.8)
**Laboratory**	
**Cryocrit,** %, median (range)	**5** (1–16)
**Immunochemical type**	
**• II,***n* (%)	**96** (94.1)
**• III,***n* (%)	**6** (5.9)
**Rheumatoid factor,** (IU/ml, ≤20), median (range)	**174** (20–4,470)
**Immunoglobulins**	
**• IgM** (mg/dl, 40–230), median (range)	**255** (17–1,273)
**• IgG** (mg/dl, 700–1,600), median (range)	**1,180** (151–2,220)
**Complement fraction**	
**• C1q** (mg/dl, 21–39), median (range)	**36.5** (25–141)
**• C3** (mg/dl, 90–180), median (range)	**97.8** (4.1-228)
**• C4** (mg/dl, 10–40), median (range)	**3.15** (1–22)
**ALT** (IU/L, 30–65), median (range)	**49** (19–265)
**GGT** (IU/L, 5–55), median (range)	**57.5** (31–145)
**Peripheral lymphocytogram**	
**CD3** (%, 72 ± 10.2), median (range)	**74** (36–89)
**CD20** (%, 10.2 ± 5.4), median (range)	**16** (5–73)
**Symptoms**	
**Palpable purpura,***n* (%)	**85** (83.3)
**Weakness,***n* (%)	**61** (59.8)
**Arthralgias/nonerosive arthritis**, *n* (%)	**66** (64.7)
**Cutaneous ulcers,***n* (%)	**40** (39.2)
**Peripheral neuropathies,***n* (%)	**36** (35.3)
**Renal disease,***n* (%)	**24** (23.5)
**Membranoproliferative glomerulonephritis,***n* (%)	**19** (79.2)
**Nephrotic syndrome,***n* (%)	**5** (20.8)

HCV gt-1 was detected in 51 patients (50%), HCV gt-2 in 49 (48.1%), and HCV gt-3 in the remaining two patients. The median age was 68 years, ranging from 44 to 80 years. Female gender greatly prevailed (78.4%), and the median MCG disease duration was 7.5 years (1 to 26 years). Twenty-four (23.5%) patients had a history of blood or blood-products transfusions. In the remainder, the source of the HCV infection was unidentified. Overall, liver biopsy, performed in all patients at the time of study inclusion, revealed a histologic picture compatible with chronic active liver disease.

To evidence the *in vivo* phenotypic changes, peripheral blood lymphocytes, obtained on the day of liver biopsy, were analyzed with flow cytometry. Clinically, cutaneous ulcers, peripheral neuropathy, and renal disease (including glomerulonephritis and/or nephrotic syndrome) were confirmed in 40 (39.2%), 36 (35.3%), and 24 (23.5%) patients, respectively.

**Figure 2:**
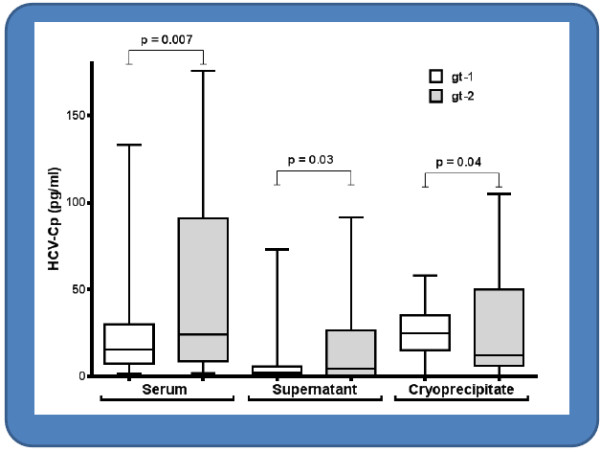
Comparison of HCV-Cp levels in unfractionated sera, cryoprecipitates, and supernatants in 51 MCG patients infected with HCV gt-1 and 49 MCG patients infected with HCV gt- 2.

Compared with gt-2 patients, those with gt-1 had significantly lower HCV-Cp median concentrations, both in unfractionated serum samples (15.6 pg/ml versus 24.1 pg/ml; *P* = 0.007) and in the supernatants (2.3 pg/ml versus 4.6 pg/ml; *P* = 0.03). Conversely, HCV-Cp enrichment in the cryoprecipitates was significantly higher in gt-1 patients than in gt-2 patients (25.0 pg/ml versus 12.0 pg/ml, *P* = 0.04) (Figure 2). Differences in selective enrichment of cold-precipitated HCV-Cp in gt-1 as compared with gt-2 patients were also confirmed in samples normalized for cryocrit (*P* < 0.002) and for cryoprecipitable IgM (*P* = 0.009), whereas no difference was achieved when samples were normalized for RF activity (*P* = 0.48) and for protein concentration (*P* = 0.33).

**Figure 3:**
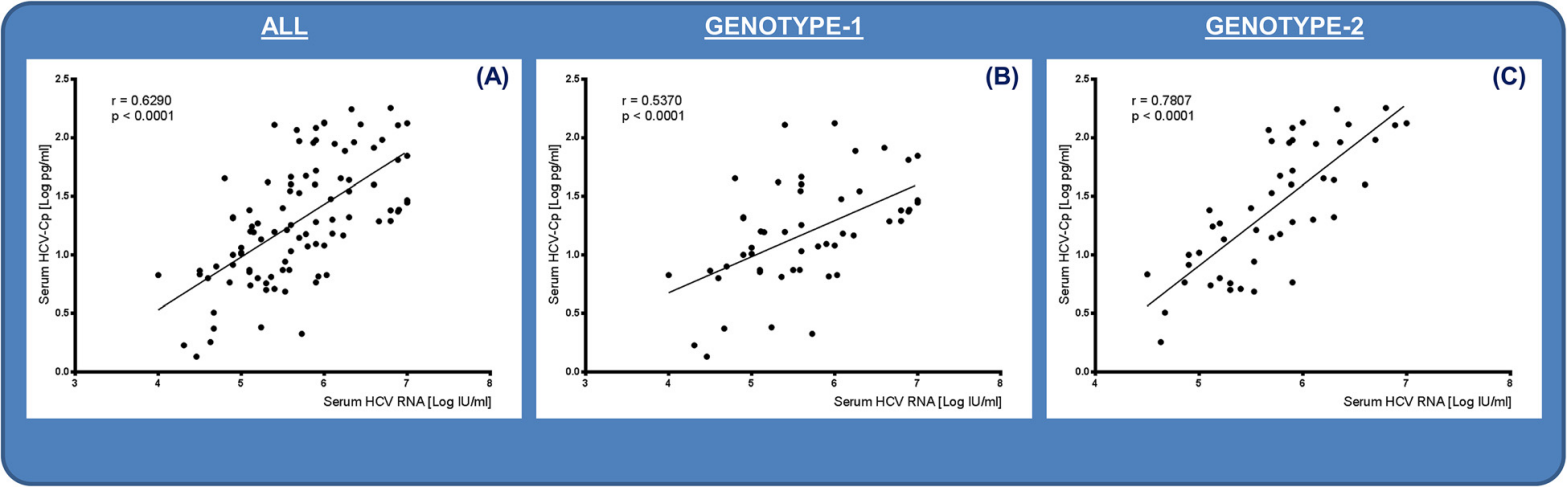
**Correlation between HCV-Cp and HCV RNA concentrations in all unfractionated serum samples (A) and in those grouped according to viral genotype 1 (B) and genotype 2 (C).** Data on the axes are log scaled.

These data suggested that the cold-dependent insolubility of HCV-Cp was gt-dependent, implying that the dynamics of the cold-precipitation process may be different between viral gts. As depicted in Figure 3, individual HCV-Cp levels correlated well with those of HCV RNA, both in the unfractionated serum samples evaluated in the complete series and as a function of gts type. The *r* coefficient for the complete series was 0.6290 (95% CI, 0.4919 to 0.7356: *P* < 0.0001) (Figure 3A). In HCV gt-1 (Figure 3B) and gt-2 (Figure 3C) patients, a positive statistically significant correlation was determined (*r* = 0.5370 (95% CI, 0.3068 to 0.7079: *P* < 0.0001) and 0.7807 (95% CI, 0.6362 to 0.8724: *P* < 0.0001), respectively].

Because all samples came from patients who were treatment-naïve (that is, not treated with any antiviral or immunosuppressive therapy, the HCV RNA-to-HCV-Cp equivalence could be calculated. In the linear regression analysis, HCV-Cp (log pg/ml) = 0.4791 × HCV RNA (log IU/ml) – 1.5111 for gt-1 patients and 0.6794 × HCV RNA (log IU/ml) – 2.7916 for gt-2 patients. Thus, 1 pg of HCV-Cp per milliliter of serum sample was equivalent to 1,425 IU and 12,850 IU of gt-1 and gt-2 HCV RNA/ml, respectively. For individual samples, this correspondence varied substantially around the average, ranging from 899 to 5,643 IU of gt-1 HCV RNA and from 8,333 to 25,106 IU of gt-2 HCV RNA. This disagreement in the ratio of HCV RNA and HCV-Cp in HCV gt-1 and gt-2 patients likely reflects the different amounts of core protein not associated with complete viral genome, which may influence the cryoprecipitation process.

**Figure 4:**
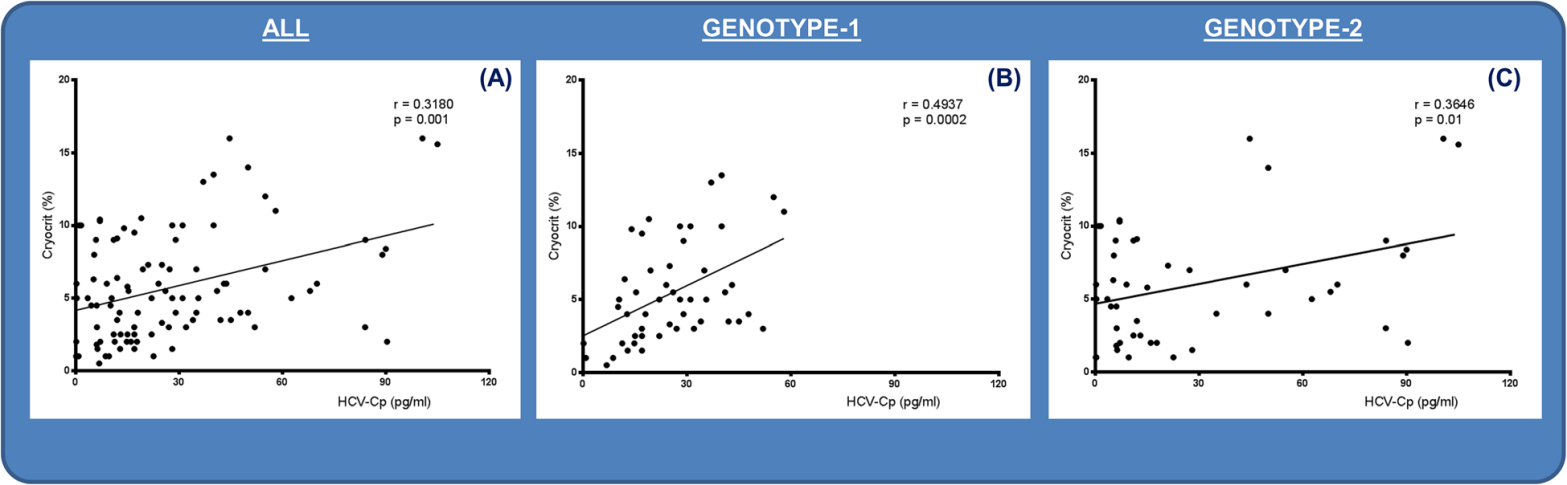
Correlation between HCV-Cp levels and cryocrit values in all patients (A) and in those grouped according to viral genotype 1 (B) and genotype 2 (C).

We also asked whether cold-precipitated HCV-Cp strictly correlated with the amount of cryoprecipitates (Figure 4). When assessed in the complete series, a correlation coefficient of 0.3180 (95% CI, 0.1239 to 0.4887: *P* = 0.001) was determined (Figure 4A). The correlation was stronger in gt-1 than in gt-2 patients (*r* = 0.4937 (95% CI, 0.2446 to 0.6817; *P* = 0.0002) and 0.3646 (95% CI, 0.0928 to 0.5858; *P* = 0.01)] (Figure 4B,C).

**Figure 5:**
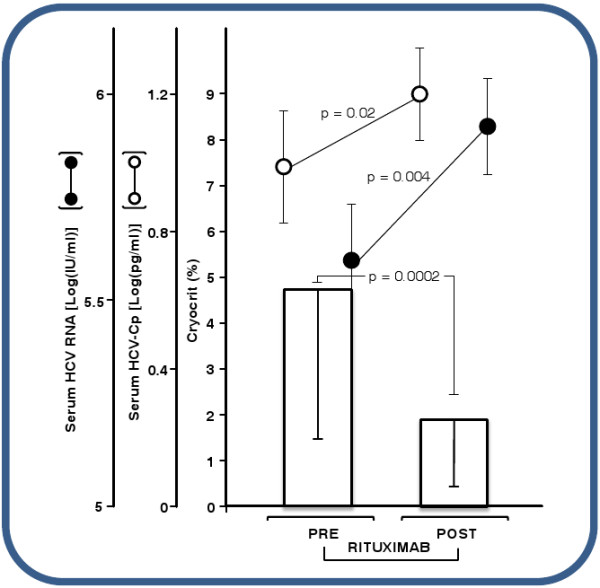
Effect of four-dose rituximab treatment on HCV RNA, HCV-Cp, and cryocrit in 16 patients with HCV-related MCG who were unresponsive to a combination of pegylated interferon-α and ribavirin.

Figure 5 summarizes the results of a four-dose RTX (schedule A) treatment plan in 16 MCG patients unresponsive to combined pIFNα plus RBV antiviral therapy. RTX therapy dramatically reduced cryoprecipitation, resulting in a decrease in the mean cryocrit from 4.8% ± 3.2% at baseline to 1.9% ± 1.4% (*P* = 0.0002) at the end of the last infusion. In step with the decrement in cryocrit levels, HCV-Cp and HCV RNA concentrations were significantly altered. HCV-Cp increased from 1.0 ± 0.18 log pg/ml at baseline to 1.2 ± 0.12 log pg/ml (*P* = 0.02), accompanied by a simultaneous sustained increment in HCV RNA, from 5.6 ± 0.2 log IU/ml to 5.9 ± 0.1 log IU/ml (*P* = 0.004).

**Figure 6:**
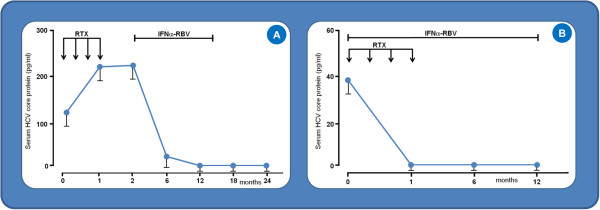
HCV-Cp concentrations under treatment schedules comprising rituximab followed by pegylated interferon-α and ribavirin (A) and the simultaneous administration of these drugs (B).

The dynamics of HCV-Cp in relation to therapeutic schedules combining RTX, pIFNα, and RBV were explored (Figure 6). In schedule B, RTX was administered once a week for 1 month, followed by pIFNα/RBV for 48 weeks. Schedule C consisted of the simultaneous administration of RTX/pIFNα/RBV. In patients treated with the former schedule, HCV-Cp levels increased in parallel with RTX infusions but declined to unmeasurable levels during subsequent pIFNα/RBV administration (Figure 6A). Conversely, in patients concomitantly treated with RTX, pIFNα, and RBV, a rapid and vigorous decline occurred in HCV-Cp (Figure 6B).

## Discussion

The present data confirm and expand our previous results, which identified HCV-Cp as a constitutive component of cryoglobulins [22]. By using a fully automated, highly sensitive chemiluminescence immunoassay for the determination of total HCV protein, we were able to overcome the shortcomings of conventional core antigen assays [24]. The intrinsic performance characteristics of this novel method include its sensitivity and specificity, even with thick and sticky cold-precipitating samples. HCV core protein was detected in the blood of 100% of the patients in our series and consistently precipitated by cold separation. By contrast, in none of the samples from patients with HCV-negative cryoglobulinemic vasculitis did the assay yield positive results.

Given the potential impact on the clinical and biologic features of MCG, several studies have focused on the quantitative relation between HCV-Cp and HCV RNA [25,27,28,33,34]. We found a direct relation between circulating HCV-Cp and serum HCV RNA levels in MCG patients, suggesting that the HCV-Cp concentration reflects viral replication. However, HCV-Cp and HCV RNA titers in serum are neither directly equivalent nor simply stoichiometrically related; thus, changes in either one are likely indicative of a change in biologic status, as reported for the HCV-infected general population [35]. RNA-free Cp-containing structures may be present in serum; however, although the serum level of HCV-Cp mirrors its intrahepatic concentration, it cannot be strictly considered a direct marker of viral replication [36].

Because the structure of HCV particles is not precisely known, the ratio of HCV RNA to Cp cannot be accurately determined. It has been estimated that 1 pg of HCV-Cp contains 130,000 HCV genome molecules [37]. In our study, HCV RNA in gt-1 and gt-2 MCG patients never exceeded 6,000 IU and 26,000 IU per picogram Cp, respectively, indicating a remarkable differences between viral gts. Because each virion presumably contains a single genome molecule and a constant number of capsomers [38], our results point to different amounts of Cp associated with HCV RNA-free structures among HCV gts. These gt-dependent differences support the estimated average association of only 3.4% (gt-1) and 35% (gt-2) of the total pool of Cp with the viral genome. Thus, in MCG patients, an overproduction of HCV-Cp by infected hepatocytes and the release of the protein as RNA-free core structures into the blood, where it acts as a decoy for the immune system, can be envisaged as the major mechanism underlying the formation of ICs with cryoprecipitating properties.

However, the reasons for the difference in HCV-Cp concentrations between HCV gts remains unclear, as already reported [39,40]. Although it could be speculated that the detection of HCV-Cp in MCG patients primarily reflects core antigen masked within antigen-antibody ICs, the analytic process is expected to dissociate these complexes. Nonetheless, supporting this assumption is the significantly greater enrichment in gt-1 patients of both cold-precipitating material with IgM molecules showing RF activity and HCV-Cp, as in this form of MCG, the RNA-to-Cp ratio is higher. It is well known that the genetic variability of HCV affects the metabolism of infected liver cells and influences the outcome of infection [41]. In addition, the RNA chaperone activity of core protein includes genomic RNA dimerization, which may constitute the regulatory switch determining translation/replication versus replication/packaging of viral RNA [42]. It has also been reported that the altered proteins in the different gts are similar, but a single amino acid change could modulate the interactions of core protein with virus and/or host proteins, strongly influencing its stability. Many of these polymorphisms may, in turn, predict RNA structures and could differentially regulate the metabolic processes that give rise to different disease conditions [43,44]. A single substitution within the HCV core-antigen sequence (A48T) reduces the sensitivity of a commonly used chemiluminescence enzyme-linked immune assay [43]. Despite the genetic variations between gts, a key viral sequence may help to predict Cp levels in MCG.

The host component interactions that explain the higher propensity to cold precipitation in gt-1 patients support the differential regulation of HCV-Cp transcription and translation within the two major HCV gts in MCG patients. If confirmed, it would distinguish individuals with HCV-related MCG from the HCV-infected general population, in which the viral genotype has no significant effect on the RNA-to-HCV Cp ratio [25,26,35,39,40,45-47].

By using the Architect assay to measure HCV-Cp in cryoprecipitates, we obtained evidence for a direct relation between the levels of cold-precipitated protein and the cryocrit, and thus were able to establish that the Cp concentration directly influences the amount of cold-precipitation. Again, a viral gt dependence could be demonstrated, in that the correlation was higher in gt-1 than in gt-2 patients. The underlying explanation for these findings is as yet unknown, but it may be that Cp does not increase in parallel with the release of HCV RNA and that the kinetics of ICs formation as well as the clearance of ICs from the circulation differ among the HCV gts. However, despite discrepancies in HCV core dynamics, organ-related damage, including nephropathies and neuropathies, is the same in MCG gt-1 and gt-2 patients, suggesting that factors other than Cp adversely affect clinical outcome.

Clinical studies have established HCV-Cp thresholds for use in tailoring the treatment of chronic HCV infection with combined pIFN-α/RBV. Quantitative measurement of total HCV-Cp may also be used during and after treatment to assess the response to therapy [27,37,40,48]. The assessment of HCV-Cp kinetics during treatment was recently shown to aid in understanding the mechanisms of action of antiviral drugs [49]. However, antiviral treatment only partially interferes with the B-cell activities responsible for the persistent production of cryoglobulins, because cryoglobulinemic vasculitis persisted in one series of patients treated successfully for HCV infection [50] and who relapsed, despite the achievement of a biologic response by another group of treated patients [51].

The introduction of RTX, a chimeric monoclonal antibody directed to CD20 antigen, has undoubtedly improved the therapeutic results in MCG by inducing *in vivo* B-cell depletion [32,52]. We previously demonstrated that RTX administration causes a substantial reduction in the cryocrit percentage, but also a significant increment of HCV RNA [32]. This was also observed in the RTX-treated patients in the present study, based on measurements of HCV-Cp levels. In this group, despite the cryocrit decrement, a prompt increase in serum HCV-Cp occurred. Enhanced core antigenemia and a parallel increase in viremia in the presence of RTX treatment are likely the result of infectious HCV particles released from lysed B cells, as demonstrated elsewhere [53]. They are, indeed, potentially harmful features that may lead to further activation of the immune system, resulting in clonal expansions of B cells with remarkable stability and functional as well as biologic autonomy. By contrast, a prompt clinical response, accompanied by the effective clearance of HCV-Cp and HCV RNA, was induced by treating patients simultaneously with RTX and pIFN-α/RBV.

## Conclusions

Our findings demonstrate that (a) HCV-Cp levels are closely related to the amount of cryoprecipitate; (b) HCV-Cp cryoprecipitation is gt-dependent; (c) the production of HCV-Cp associated with HCV RNA-free structures is considerably greater in gt-1 rather than gt-2 MCG patients; (d) a significant increment of HCV-Cp occurs after RTX-induced B-cell depletion. Nevertheless, the implications of these results on the structural and clinical features of MCG await further studies in a larger cohort of patients, including more of type III MCG, that in almost 2% of cases can spontaneously switch from type II MCG with significant changes of clinical manifestations, normalization of C4 serum levels, and lowering of RF activity, as recently demonstrated by our group in a 15-year prospective study [54].

In principle, given the strong relation between HCV-Cp and cold-precipitated proteins, these findings prove that HCV-Cp determination can be favorably applied as a supplemental test in monitoring the variegate clinical features of MCG vasculitis as well as response to antiviral treatment of these patients. It can be assumed that high production of HCV RNA-free core structures may contribute to the dysregulation of adaptive immune response, resulting in increased risk of uncontrolled lymphoproliferation in these patients [16].

## Abbreviations

B-NHL: B-cell non-Hodgkin lymphoma; CDR-3: complementarity determining region-3; CV: coefficient of variation; gC1q-R: globular domain of the C1q receptor; gt: genotype; HCV: hepatitis C virus; HCV-Cp: hepatitis C virus core protein; IC: immune complex; KS test: Kolmogorov-Smirnov test; MCG: mixed cryoglobulinemia; moAb: monoclonal antibody; PBMC: peripheral blood mononuclear cell; PCR: polymerase chain reaction; pIFN-α: pegylated interferon alpha; RBV: ribavirin; RF: rheumatoid factor; RTX: rituximab.

## Competing interests

The authors declare that they have no competing interests.

## Authors’ contributions

SR: conception and design, data collection and analysis, manuscript writing, and final approval of the manuscript; DS: conception and design, financial support, manuscript writing, final approval of manuscript; MAM: data collection and analysis, critical revision, and final approval of the manuscript; AV: data collection and analysis, critical revision, and final approval of the manuscript; FP: data collection and analysis, critical revision, and final approval of the manuscript; GL: data collection and analysis, critical revision, and final approval of the manuscript; SS data collection and analysis, critical revision, and final approval of the manuscript; FD: data collection and analysis, critical revision, and final approval of the manuscript. All authors read and approved the final manuscript.

## References

[B1] Sansonno D Dammacco F Hepatitis C virus, cryoglobulinaemia, and vasculitis: immune complex relations Lancet Infect Dis 2005 5 227 236 10.1016/S1473-3099(05)70053-0 15792740

[B2] Jacobson IM Cacoub P Dal Maso L Harrison SA Younossi ZM Manifestations of chronic hepatitis C virus infection beyond the liver Clin Gastroenterol Hepatol 2010 8 1017 1029 10.1016/j.cgh.2010.08.026 20870037

[B3] McFarlane IG Immunological abnormalities and hepatotropic viral infections Clin Exp Immunol 1992 87 337 339 1531946 10.1111/j.1365-2249.1992.tb02998.x PMC1554324

[B4] Sansonno D Iacobelli AR Cornacchiulo V Lauletta G Distasi MA Gatti P Dammacco F Immunochemical and biomolecular studies of circulating immune complexes isolated from patients with acute and chronic hepatitis C virus infection Eur J Clin Invest 1996 26 465 475 10.1046/j.1365-2362.1996.162317.x 8817160

[B5] Chen M Sallberg M Sonnerborg A Weiland O Mattsson L Jin L Birkett A Peterson D Milich DR Limited humoral immunity in hepatitis C virus infection Gastroenterology 1999 116 135 143 10.1016/S0016-5085(99)70237-4 9869611

[B6] Ferri C La Civita L Zignego AL Extrahepatic manifestations of hepatitis C virus infection Ann Intern Med 1996 125 344 10.7326/0003-4819-125-4-199608150-00018 8678401

[B7] Dammacco F Sansonno D Piccoli C Tucci FA Racanelli V The cryoglobulins: an overview Eur J Clin Invest 2001 31 628 638 10.1046/j.1365-2362.2001.00824.x 11454019

[B8] Zignego AL Ferri C Giannini C La Civita L Careccia G Longombardo G Bellesi G Caracciolo F Thiers V Gentilini P Hepatitis C virus infection in mixed cryoglobulinemia and B-cell non-Hodgkin’s lymphoma: evidence for a pathogenetic role Arch Virol 1997 142 545 555 10.1007/s007050050100 9349300

[B9] Sansonno D De Vita S Cornacchiulo V Carbone A Boiocchi M Dammacco F Detection and distribution of hepatitis C virus-related proteins in lymph nodes of patients with type II mixed cryoglobulinemia and neoplastic or non-neoplastic lymphoproliferation Blood 1996 88 4638 4645 8977256

[B10] Sansonno D De Vita S Iacobelli AR Cornacchiulo V Boiocchi M Dammacco F Clonal analysis of intrahepatic B cells from HCV-infected patients with and without mixed cryoglobulinemia J Immunol 1998 160 3594 3601 9531323

[B11] Vallat L Benhamou Y Gutierrez M Ghillani P Hercher C Thibault V Charlotte F Piette JC Poynard T Merle-Beral H Davi F Cacoub P Clonal B cell populations in the blood and liver of patients with chronic hepatitis C virus infection Arthritis Rheum 2004 50 3668 3678 10.1002/art.20594 15529359

[B12] Russi S Lauletta G Serviddio G Sansonno S Conteduca V Sansonno L De Re V Sansonno D T cell receptor variable beta gene repertoire in liver and peripheral blood lymphocytes of chronically hepatitis C virus-infected patients with and without mixed cryoglobulinaemia Clin Exp Immunol 2013 172 254 262 10.1111/cei.12035 23574322 PMC3628328

[B13] Sansonno D Lauletta G De Re V Tucci FA Gatti P Racanelli V Boiocchi M Dammacco F Intrahepatic B cell clonal expansions and extrahepatic manifestations of chronic HCV infection Eur J Immunol 2004 34 126 136 10.1002/eji.200324328 14971038

[B14] De Re V Sansonno D Simula MP Caggiari L Gasparotto D Fabris M Tucci FA Racanelli V Talamini R Campagnolo M Geremia S Dammacco F De Vita S HCV-NS3 and IgG-Fc crossreactive IgM in patients with type II mixed cryoglobulinemia and B-cell clonal proliferations Leukemia 2006 20 1145 1154 10.1038/sj.leu.2404201 16617326

[B15] Racanelli V Sansonno D Piccoli C D’Amore FP Tucci FA Dammacco F Molecular characterization of B cell clonal expansions in the liver of chronically hepatitis C virus-infected patients J Immunol 2001 167 21 29 10.4049/jimmunol.167.1.21 11418627

[B16] Dammacco F Sansonno D Piccoli C Racanelli V D’Amore FP Lauletta G The lymphoid system in hepatitis C virus infection: autoimmunity, mixed cryoglobulinemia, and overt B-cell malignancy Semin Liver Dis 2000 20 143 157 10.1055/s-2000-9613 10946420

[B17] Santer DM Ma MM Hockman D Landi A Tyrrell DL Houghton M Enhanced activation of memory, but not naive. B cells in chronic hepatitis C virus-infected patients with cryoglobulinemia and advanced liver fibrosis PLoS One 2013 8 e68308 10.1371/journal.pone.0068308 23840845 PMC3695964

[B18] Sansonno D Tucci FA Ghebrehiwet B Lauletta G Peerschke EI Conteduca V Russi S Gatti P Sansonno L Dammacco F Role of the receptor for the globular domain of C1q protein in the pathogenesis of hepatitis C virus-related cryoglobulin vascular damage J Immunol 2009 183 6013 6020 10.4049/jimmunol.0902038 19828637 PMC2794553

[B19] Wei G Yano S Kuroiwa T Hiromura K Maezawa A Hepatitis C virus (HCV)-induced IgG-IgM rheumatoid factor (RF) complex may be the main causal factor for cold-dependent activation of complement in patients with rheumatic disease Clin Exp Immunol 1997 107 83 88 10.1046/j.1365-2249.1997.d01-882.x 9010261 PMC1904537

[B20] Mouquet H Scheid JF Zoller MJ Krogsgaard M Ott RG Shukair S Artyomov MN Pietzsch J Connors M Pereyra F Walker BD Ho DD Wilson PC Seaman MS Eisen HN Chakraborty AK Hope TJ Ravetch JV Wardemann H Nussenzweig MC Polyreactivity increases the apparent affinity of anti-HIV antibodies by heteroligation Nature 2010 467 591 595 10.1038/nature09385 20882016 PMC3699875

[B21] Charles ED Orloff MI Nishiuchi E Marukian S Rice CM Dustin LB Somatic hypermutations confer rheumatoid factor activity in hepatitis C virus-associated mixed cryoglobulinemia Arthritis Rheum 2013 65 2430 2440 10.1002/art.38041 23754128 PMC4026862

[B22] Sansonno D Lauletta G Nisi L Gatti P Pesola F Pansini N Dammacco F Non-enveloped HCV core protein as constitutive antigen of cold-precipitable immune complexes in type II mixed cryoglobulinaemia Clin Exp Immunol 2003 133 275 282 10.1046/j.1365-2249.2003.02204.x 12869035 PMC1808767

[B23] Yao ZQ Ray S Eisen-Vandervelde A Waggoner S Hahn YS Hepatitis C virus: immunosuppression by complement regulatory pathway Viral Immunol 2001 14 277 295 10.1089/08828240152716547 11792059

[B24] Morota K Fujinami R Kinukawa H Machida T Ohno K Saegusa H Takeda K A new sensitive and automated chemiluminescent microparticle immunoassay for quantitative determination of hepatitis C virus core antigen J Virol Methods 2009 157 8 14 10.1016/j.jviromet.2008.12.009 19135481

[B25] Mederacke I Wedemeyer H Ciesek S Steinmann E Raupach R Wursthorn K Manns MP Tillmann HL Performance and clinical utility of a novel fully automated quantitative HCV-core antigen assay J Clin Virol 2009 46 210 215 10.1016/j.jcv.2009.08.014 19766055

[B26] Vermehren J Susser S Berger A Perner D Peiffer KH Allwinn R Zeuzem S Sarrazin C Clinical utility of the ARCHITECT HCV Ag assay for early treatment monitoring in patients with chronic hepatitis C genotype 1 infection J Clin Virol 2012 55 17 22 10.1016/j.jcv.2012.05.008 22698697

[B27] Tedder RS Tuke P Wallis N Wright M Nicholson L Grant PR Therapy-induced clearance of HCV core antigen from plasma predicts an end of treatment viral response J Viral Hepat 2013 20 65 71 23231086 10.1111/j.1365-2893.2012.01630.x

[B28] Miedouge M Saune K Kamar N Rieu M Rostaing L Izopet J Analytical evaluation of HCV core antigen and interest for HCV screening in haemodialysis patients J Clin Virol 2010 48 18 21 10.1016/j.jcv.2010.02.012 20233674

[B29] Echevarria JM Avellon A Jonas G Hausmann M Vockel A Kapprell HP Sensitivity of a modified version of the ARCHITECT Anti-HCV test in detecting samples with immunoblot-confirmed, low-level antibody to hepatitis C virus J Clin Virol 2006 35 368 372 10.1016/j.jcv.2005.11.006 16406797

[B30] The French METAVIR Cooperative Study Group Intraobserver and interobserver variations in liver biopsy interpretation in patients with chronic hepatitis C Hepatology 1994 20 15 20 8020885

[B31] Brouet JC Clauvel JP Danon F Klein M Seligmann M Biologic and clinical significance of cryoglobulins: a report of 86 cases Am J Med 1974 57 775 788 10.1016/0002-9343(74)90852-3 4216269

[B32] Sansonno D De Re V Lauletta G Tucci FA Boiocchi M Dammacco F Monoclonal antibody treatment of mixed cryoglobulinemia resistant to interferon alpha with an anti-CD20 Blood 2003 101 3818 3826 10.1182/blood-2002-10-3162 12506023

[B33] Kesli R Polat H Terzi Y Kurtoglu MG Uyar Y Comparison of a newly developed automated and quantitative hepatitis C virus (HCV) core antigen test with the HCV RNA assay for clinical usefulness in confirming anti-HCV results J Clin Microbiol 2011 49 4089 4093 10.1128/JCM.05292-11 21940466 PMC3233016

[B34] Kuo YH Chang KC Wang JH Tsai PS Hung SF Hung CH Chen CH Lu SN Is hepatitis C virus core antigen an adequate marker for community screening? J Clin Microbiol 2012 50 1989 1993 10.1128/JCM.05175-11 22461676 PMC3372126

[B35] Park Y Lee JH Kim BS Kim Do Y Han KH Kim HS New automated hepatitis C virus (HCV) core antigen assay as an alternative to real-time PCR for HCV RNA quantification J Clin Microbiol 2010 48 2253 2256 10.1128/JCM.01856-09 20351215 PMC2884480

[B36] Descamps V Op De Beeck A Plassart C Brochot E Francois C Helle F Adler M Bourgeois N Degre D Duverlie G Castelain S Strong correlation between liver and serum levels of hepatitis C virus core antigen and RNA in chronically infected patients J Clin Microbiol 2012 50 465 468 10.1128/JCM.06503-11 22162563 PMC3264181

[B37] Bouvier-Alias M Patel K Dahari H Beaucourt S Larderie P Blatt L Hezode C Picchio G Dhumeaux D Neumann AU McHutchison JG Pawlotsky JM Clinical utility of total HCV core antigen quantification: a new indirect marker of HCV replication Hepatology 2002 36 211 218 12085367 10.1053/jhep.2002.34130

[B38] Schuttler CG Thomas C Discher T Friese G Lohmeyer J Schuster R Schaefer S Gerlich WH Variable ratio of hepatitis C virus RNA to viral core antigen in patient sera J Clin Microbiol 2004 42 1977 1981 10.1128/JCM.42.5.1977-1981.2004 15131157 PMC404599

[B39] Ross RS Viazov S Salloum S Hilgard P Gerken G Roggendorf M Analytical performance characteristics and clinical utility of a novel assay for total hepatitis C virus core antigen quantification J Clin Microbiol 2010 48 1161 1168 10.1128/JCM.01640-09 20107102 PMC2849592

[B40] Alsio A Jannesson A Langeland N Pedersen C Farkkila M Buhl MR Morch K Westin J Hellstrand K Norkrans G Lagging M Early quantification of HCV core antigen may help to determine the duration of therapy for chronic genotype 2 or 3 HCV infection Eur J Clin Microbiol Infect Dis 2012 31 1631 1635 10.1007/s10096-011-1486-5 22113307

[B41] Chayama K Hayes CN Yoshioka K Moriwaki H Okanoue T Sakisaka S Takehara T Oketani M Toyota J Izumi N Hiasa Y Matsumoto A Nomura H Seike M Ueno Y Yotsuyanagi H Kumada H Factors predictive of sustained virological response following 72 weeks of combination therapy for genotype 1b hepatitis C J Gastroenterol 2011 46 545 555 10.1007/s00535-010-0358-6 21246384

[B42] Ivanyi-Nagy R Lavergne JP Gabus C Ficheux D Darlix JL RNA chaperoning and intrinsic disorder in the core proteins of Flaviviridae Nucleic Acids Res 2008 36 712 725 18033802 10.1093/nar/gkm1051 PMC2241907

[B43] Ogata S Nagano-Fujii M Ku Y Yoon S Hotta H Comparative sequence analysis of the core protein and its frameshift product, the F protein, of hepatitis C virus subtype 1b strains obtained from patients with and without hepatocellular carcinoma J Clin Microbiol 2002 40 3625 3630 10.1128/JCM.40.10.3625-3630.2002 12354856 PMC130847

[B44] Tang X Wagoner J Negash A Austin M McLauchlan J Hahn YS Rosen HR Polyak SJ Functional characterization of core genes from patients with acute hepatitis C virus infection J Infect Dis 2010 201 912 922 10.1086/650699 20170366 PMC2827820

[B45] Saeed M Suzuki R Kondo M Aizaki H Kato T Mizuochi T Wakita T Watanabe H Suzuki T Evaluation of hepatitis C virus core antigen assays in detecting recombinant viral antigens of various genotypes J Clin Microbiol 2009 47 4141 4143 10.1128/JCM.01437-09 19812276 PMC2786616

[B46] Medici MC Furlini G Rodella A Fuertes A Monachetti A Calderaro A Galli S Terlenghi L Olivares M Bagnarelli P Costantini A De Conto F Sainz M Galli C Manca N Landini MP Dettori G Chezzi C Hepatitis C virus core antigen: analytical performances, correlation with viremia and potential applications of a quantitative, automated immunoassay J Clin Virol 2011 51 264 269 10.1016/j.jcv.2011.05.003 21621454

[B47] Veillon P Payan C Picchio G Maniez-Montreuil M Guntz P Lunel F Comparative evaluation of the total hepatitis C virus core antigen, branched-DNA, and amplicor monitor assays in determining viremia for patients with chronic hepatitis C during interferon plus ribavirin combination therapy J Clin Microbiol 2003 41 3212 3220 10.1128/JCM.41.7.3212-3220.2003 12843066 PMC165326

[B48] Loggi E Cursaro C Scuteri A Grandini E Panno AM Galli S Furlini G Bernardi M Galli C Andreone P Patterns of HCV-RNA and HCV core antigen in the early monitoring of standard treatment for chronic hepatitis C J Clin Virol 2013 56 207 211 23245628 10.1016/j.jcv.2012.11.012

[B49] Akuta N Suzuki F Hirakawa M Kawamura Y Yatsuji H Sezaki H Suzuki Y Hosaka T Kobayashi M Saitoh S Arase Y Ikeda K Kumada H Amino acid substitutions in the hepatitis C virus core region of genotype 1b affect very early viral dynamics during treatment with telaprevir, peginterferon, and ribavirin J Med Virol 2010 82 575 582 10.1002/jmv.21741 20166188

[B50] Dammacco F Sansonno D Therapy of HCV-related mixed cryoglobulinemia N Engl J Med 2013 369 1035 1045 10.1056/NEJMra1208642 24024840

[B51] Landau DA Saadoun D Halfon P Martinot-Peignoux M Marcellin P Fois E Cacoub P Relapse of hepatitis C virus-associated mixed cryoglobulinemia vasculitis in patients with sustained viral response Arthritis Rheum 2008 58 604 611 10.1002/art.23305 18240235

[B52] Zaja F De Vita S Mazzaro C Sacco S Damiani D De Marchi G Michelutti A Baccarani M Fanin R Ferraccioli G Efficacy and safety of rituximab in type II mixed cryoglobulinemia Blood 2003 101 3827 3834 10.1182/blood-2002-09-2856 12560225

[B53] Stamataki Z Tilakaratne S Adams DH McKeating JA Rituximab treatment in hepatitis C infection: an in vitro model to study the impact of B cell depletion on virus infectivity PLoS One 2011 6 e25789 10.1371/journal.pone.0025789 21991396 PMC3184166

[B54] Lauletta G Russi S Conteduca V Sansonno L Dammacco F Sansonno D Impact of cryoglobulinemic syndrome on the outcome of chronic hepatitis C virus infection: a 15-year prospective study Medicine (Baltimore) 2013 92 245 256 10.1097/MD.0b013e31829d2abc PMC4553977 23982056

